# The Role of Pseudocereals in Celiac Disease: Reducing Nutritional Deficiencies to Improve Well-Being and Health

**DOI:** 10.1155/2022/8502169

**Published:** 2022-02-09

**Authors:** Carolina Caeiro, Caroline Pragosa, Marisa Carreira Cruz, Cidália Daniela Pereira, Sónia Gonçalves Pereira

**Affiliations:** ^1^School of Health Sciences, Polytechnic of Leiria, Leiria, Portugal; ^2^Center for Innovative Care and Health Technology, Polytechnic of Leiria, Leiria, Portugal

## Abstract

Celiac disease or gluten-dependent enteropathy is a chronic autoimmune pathology triggered by dietary gluten in genetic predisposed individuals, mediated by transglutaminase 2 IgA autoantibodies and associated with a deteriorating immune and inflammatory response. This leads to intestinal villous atrophy, impairing the intestinal mucosa structure and function of secretion, digestion, and absorption. The result is macro- and micronutrient deficiency, including fat soluble vitamins and minerals, and a consequent nutritional status depletion. A lifelong gluten-free diet is the only available treatment for celiac patients in order to assure normal intestinal mucosa and remission of gastrointestinal symptoms. However, a gluten-free diet can itself cause other nutritional deficiencies due to its restrictive nature regarding gluten-containing cereals. A group of gluten-free cereals, known as pseudocereals, is increasingly recognized as valuable options for gluten-free diets due to their high nutritional value. Amaranth, quinoa, millet, and buckwheat are examples of gluten-free nutrient-dense grains that can be used as alternatives to the conventional gluten-containing grains and improve the variety and nutritional quality of the celiac diet. Current work reviews the nutritional pitfalls of a gluten-free diet and analyses how pseudocereals can contribute to revert those deficiencies and optimize the nutritional value of this mandatory diet for the celiac population.

## 1. Introduction

Celiac disease (CD) is an autoimmune condition that results in intestinal mucosal injury caused by dietary gluten—a generic term specific for proteins (prolamins) found in the endosperm of some cereals, specifically wheat (gliadins), rye (secalins), and barley (hordeins) [1]. This pathology is a worldwide problem that despite being first described in countries with Caucasian population is also common in other parts of the world. The global seroprevalence of CD is 1.4%, and the prevalence of biopsy-proven CD is 0.7%, varying according to age, gender, and geography. The most affected countries (76 to 100 percentile) are Mexico, Portugal, Sweden, Czech Republic, Turkey, Algeria, Saudi Arabia, India, and Malaysia. Regarding gender, a systemic review and meta-analysis revealed a clear gender discrepancy in patients with undiagnosed CD with women more frequently affected. However, more evidence is needed to explore the gender differences observed in CD and the underpinning biological mechanisms [2]. Concerning age, there is a lack of robust evidence to reveal conclusive data [3].

The only treatment available for CD is adherence to a gluten-free diet (GFD). However, the patient often struggles with following a GFD or in choosing adequate nutritional food. This diet can result in nutritional deficiency or oversufficiency (for example of fiber or saturated fat, respectively); thus, it is valuable that patients are regularly followed by a nutritionist [4]. Even though a GFD limits the ingestion of tubers, cereals, and its derivatives, pseudocereals, a group of nongrass and naturally gluten-free plants, can decrease these limitations and enrich the GFD [5].

This literature review focuses on foreseeing the use of pseudocereals in a GFD in order to increase dietary variety and quality for CD patients.

## 2. Physiopathology of Celiac Disease

Besides genetic predisposition and a specific immune response, CD development is dependent on gluten exposure that acts as a trigger [6]. Gluten is composed of gliadins (monomeric proteins) and glutenins (polymeric protein aggregates), rich in proline and glutamine peptides (soluble in alcoholic aqueous solutions), resistant to degradation by intestinal lumen endopeptidases and thereby undigested in the gastrointestinal tract [7].

Once ingested, gluten is partially cleaved into gliadin peptides that pass through the intestinal mucosa epithelial barrier due to increased permeability [8], caused by the inflammatory innate immune response. In the lamina propria (the intermediate connective tissue layer of the intestinal mucosa) occurs an important step in CD pathogenesis, gliadin deamidation by the tissue transglutaminase enzyme, which launches the activation of the adaptive immune system [9]. This adaptive immune response against gliadin involves antigen-presenting cells such as macrophages, dendritic cells, and B cells [7].

The innate immune response to gliadin occurs in the intestinal mucosa epithelial layer and increases the release of cytokines, namely interleukin-15 (IL-15), produced by enterocytes, macrophages, and dendritic cells. This results in intraepithelial leukocyte differentiation into CD8+ cytotoxic T cells that express the marker for natural killer NK-G2D cells causing epithelial cell apoptosis [10]. The accumulation of all these inflammatory mediators leads to intestinal mucosal injury that manifests through flattening of the villi and elongation of the crypts, the histological alterations characteristic of CD [11].

### 2.1. Celiac Disease Subtypes

Celiac disease can be divided into two main types, symptomatic (or classic) and asymptomatic [12]. The classical subtype or symptomatic applies to cases with classical characteristics of CD (chronic diarrhoea, abdominal pain and distension, asthenia, and malabsorption), whereas in the atypical or asymptomatic subtype, the gastrointestinal symptoms are absent or occur to a lesser degree and more prominent manifestations are common such as anemia, osteoporosis, lower stature, infertility, and neurological problems [12, 13].

## 3. Celiac Disease Treatment

The basis of CD treatment is a lifelong strict adherence to a GFD [14]. For most patients, adherence to a GFD guarantees intestinal mucosa cicatrisation and gastrointestinal symptoms remission [15] in approximately four weeks [16].

A GFD also reduces future health problems like osteoporosis and improves mental well-being and quality of life [17]. Improvement in symptoms, like diarrhoea resolution and reduction of abdominal pain and distension, normally precedes serological normalization of tissue transglutaminase antibodies (which can take months or up to a year) followed by histological normalization [16].

Though the maximum nontoxic quantity of gluten consumption for CD patients is unknown, <10 mg/per day is considered a safe amount to prevent intestinal lesions [18]. To label foods as gluten-free, the Food and Drug Administration (FDA) requires these to have a gluten content of <20 parts/per million [19].

### 3.1. Challenges of a Gluten-Free Diet

Following a GFD implies absolute removal of dietary gluten, obviously excluding not only all ingredients that naturally contain gluten from celiac patients' diets but also all gluten sources in food that may derive from cross contamination during processing (e.g., modified starch and dietary fiber with no information of its cereal source), which can be demanding. The European Commission regulation n. 41/2009, of 20 January 2009, clarified food products composition and labeling regarding gluten content that can help patients select the right dietary options [20]. However, cross contamination during food preparation, particularly in restaurant and food service industries, still is a very present-day and socially limiting reality for these patients [21]. Another relevant challenge is the cost associated with gluten-free food. Even though its availability and variety have increased in recent years, its cost remains significantly higher than the equivalent gluten-containing options [22]. Adherence to treatment may be a distressing task due of its high cost, adding up as another social isolation factor for these patients, apart from their fear of food contamination in restaurants and food service industries [23].

As for dietary diversity in a GFD, corn, potato, and rice comprise the food options more consumed by celiac patients and the most common food sources in the preparation of gluten-free products by the food industry. This restriction can originate nutritional imbalances [4], usually present upon diagnosis, that can persist after the adoption of a GFD due to its incapacity to suppress all nutritional demands [24]. There are other naturally gluten-free cereals and derivatives like buckwheat, millet, quinoa, bulgur, sorghum, and amaranth that if included may diversify a GFD and avoid macronutrient, vitamin, and mineral deficiencies [8].

Apart from these cereals and derivatives, oats can also be incorporated in a GFD, increasing the nutritional value not only due to its protein (10 to 15%) and lipid (5 to 9%) content but also its high fiber, thiamine, and zinc content in comparison to conventional gluten-containing cereals like wheat, rye, and barley [25]. Oats are also associated with an increase in palatability and satiety [25]. Some studies demonstrated that celiac patients who consume oats in a long-term GFD have the same symptoms, serology, and a similar life quality or even higher of those whose diet is oat-free [26], proving to be safe for consumption [27]. Other studies state that oats also have, though to a lesser degree, prolamins, a toxic gluten component for celiac patients [28], mainly because of cross contamination during cultivation and/or production processes [29]. Thus, oats inclusion in a GFD remains controversial and should be made with precaution and vigilance, specifically through the careful analysis of food labels and should be removed from diet in case it triggers characteristic symptomology or increases CD specific antibodies [25].

## 4. Nutritional Status of a Celiac Disease Patient

Recently diagnosed adults with CD usually have lower levels of body mass and body fat percentage compared tononceliac individuals [13] as well as a lower body mass index (BMI). Although usually associated with low stature and low weight, some studies demonstrate that adult CD patients can have a normal or preobese BMI, making it harder to diagnose CD [13, 30]. Concomitantly, CD patients may have osteomalacia, osteopenia, or even osteoporosis, caused by vitamin and mineral deficiencies that lead to a low bone mineral density [13, 31].

Concerning the role of a GFD in the nutritional status of CD patients, it is commonly observed a weight gain after a period on this diet, although they remain in the same BMI category [13]. Few studies record weight loss in patients who were in a preobesity status (BMI between 25, 0–29, 5 kg/m^2^) [13, 30, 31]. Regarding the influence of this diet on bone mineral density, there is a lack of data in adult CD patients, and studies in pediatric patients concluded that CD children who followed a GFD had a lower risk of having low bone mineral density [32]. On the other hand, children who have this pathology and adopted a GFD continue to exhibit higher body free fat mass and lower body fat mass percentages than nonceliac children [33].

### 4.1. Nutritional Imbalances Inherent to Celiac Disease

The innate and adaptive immune responses activated by gluten ingestion in CD patients result in villous atrophy and crypt hyperplasia of the small intestinal mucosa [23]. These changes cause nutrient malabsorption that can lead to multiple deficiencies in fat soluble vitamins, minerals, and micronutrients [18], including iron, folic acid, vitamins A, B6, B12, D, E, K, copper, and zinc [34].

Cobalamin or B12 deficiency occurs in about 12 to 41% of celiac patients, with terminal ileum involvement [35], pancreatic insufficiency [36], and/or concomitant autoimmune gastritis, which in itself causes B12 deficiency in 10% of these patients [37]. Gastric acid releases cobalamin from food sources which then binds to the intrinsic factor [38], indispensable for its absorption in the terminal ileum via specific receptors [4]. As most CD patients develop ileum disease, B12 absorption is difficult even with a proper diet. Due to possible irreversible neurological consequences of B12 deficiency, all CD patients must have their B12 levels checked regularly to allow supplementation if necessary [18].

Patients with CD can also have deficiencies in folic acid or folate. This micronutrient, absorbed primarily in the proximal small intestine, more precisely in the jejunum [24], is reduced and methylated and then transported to the liver for posterior systemic circulation. This process is interrupted in celiac enteropathy owing to contact surface decrease between folic acid and enterocytes as a result of the villous atrophy [4]. Thereby, folic acid supplementation is recommended in cases of malabsorption or serious anemia, since serum levels may differ from intraerythrocyte levels [39].

Pyridoxine or B6 vitamin deficiency is also common in CD patients. B6 is a vital nutrient for amino acid metabolism, hemoglobin, and neurotransmitter synthesis, and also gene expression [11]. It is absorbed in the jejunum and ileum, where there is malabsorption caused by enteropathy in the case of CD patients. For this reason, it is important to monitor B6 vitamin levels in case other vitamin B complex deficiencies or if symptoms associated with its deficiency occur, like cheilosis, glossitis, estomatitis, insomnia, and peripheral neuropathies [4].

Vitamin D or calciferol has a diverse role in human physiology. Its homeostasis is crucial to maintain bone mineralization and prevent osteoporosis [40]. Vitamin D is produced mainly in the skin where it is converted from 7-dehydrocholesterol to pre-vitamin D3 by UV light [1], and only a small fraction is obtained from diet. Soluble in fat, its absorption requires intact intestinal, biliary, and pancreatic systems, and therefore, its levels can be significantly affected by enteropathies like CD [41]. Vitamin D influences calcium absorption and metabolism, with both being found at low levels in most nontreated CD patients [42]. These deficiencies can derive from malabsorption, intestinal epithelial injury, and/or dairy products nonconsumption due to concomitant lactose intolerance, which is frequent in these patients [41].

Iron is also a nutrient which may be in deficit in CD. It has essential functions in the transport and storage of oxygen, being the active centre of hemoglobin, vital for cellular respiration and oxygen transport, and mioglobin, for the storage of oxygen in the muscles [1]. Iron is mainly absorbed in the proximal small intestine, the region most affected by CD [24]. Another possible cause of iron deficiency is the loss of duodenal enterocytes, the storage site of ferritin [43]. Iron deficiency anemia is one of the most common clinical manifestations affecting up to 32% CD adults. In a recently diagnosed CD patient, a complete blood count with ferritin level quantification must be performed to rule out iron deficiency anemia [18]. Upon diagnosis of this type of anemia, CD must be considered when all other common causes have been ruled out. If a deficiency is detected, supplementation should be initiated and maintained until remission of the symptoms [42].

CD patients can also be deficient in copper, selenium, and zinc. However, supplementation is unadvised since its deficiencies reverse rapidly when patients follow a GFD [39].

### 4.2. Nutritional Imbalances Associated with a Gluten-Free Diet

A GFD is often characterized by lower fiber ingestion comparatively to an unrestricted diet [44], since gluten-free foods, frequently produced with refined starch and flours, have low fiber content [45]. Fiber deficiency can be observed upon the diagnosis as well as during a GFD, appearing to be related to poor nutritional quality of the food chosen by the patients [45].

A GFD can cause other nutritional deficiencies because of the low quality of gluten-free food and the insufficient ingestion of folate [46] that result in an imbalance between macronutrient ingestion and energy supply [4]. A study performed on CD patients from adolescence until adulthood concluded that they follow an unbalanced diet even after GFD implementation, with an inadequate ingestion of some nutrients such as fibers, calcium, and iron, along with the ingestion of a high proportion of fat as energy supply [47]. Another study performed in children demonstrated a low consumption of iron, magnesium, selenium, and vitamin B12 in CD patients in comparison to healthy children [48].

Other deficiencies may appear or even surpluses. Kulai and Rashid [49] demonstrated significant differences in gluten-free bread and pasta, foods consumed in higher frequency and quantity by these patients. Gluten-free bread has a higher fat content (more than double) and a lower protein and iron content in comparison to gluten-containing bread. Gluten-free pasta has a higher amount of carbohydrates and lower protein, fiber, folate, and iron content (a third less comparatively to regular pasta). One of the possible explanations for these deficiencies may lie in the lack of regulation to impose nutritional fortification of gluten-free food. In some countries, regulations only enforce wheat-based products to have enrichments or nutritional fortifications, excluding “special and dietetic food,” where gluten-free products are included [34]. The reduced gluten-free food nutritional fortification may increase the risk of micronutrient deficiency in seemingly healthy CD patients on a GFD, which can be a risk factor for their health and well-being [46]. This risk may also be further increased by the unawareness of CD patients regarding other food options besides the more traditional gluten-free ones like potatoes, corn, and rice. Nutritional literacy is very important for these patients. Learning the benefits of pseudocereals can be a plus, by allowing them to enrich their diets.

### 4.3. The Role of Pseudocereals in Optimizing a Gluten-Free Diet

Pseudocereals like amaranth, quinoa, millet, and buckwheat have been increasingly recognized as relevant for a GFD due to their high nutritional value. Rich in starch, fiber, and high biological value proteins, pseudocereals are also an excellent source of vitamins and minerals and for this reason have been studied as an alternative to diversify and optimize a GFD [50].

The protein bioavailability in pseudocereals is higher and superior to common cereals and of similar quality as animal protein [51]. This is relevant for the inclusion of pseudocereals in a GFD since exclusion of certain cereals from the diet may lead to loss of protein sources [52]. Studies demonstrate that amaranth and quinoa contain protein more digestible, efficient and of nutritional balance [5]. The same applies to fiber. Buckwheat fiber content, in particular, is significantly higher than that of common cereals and other pseudocereals (quinoa and amaranth). Inclusion of buckwheat in a GFD can help reduce fiber deficiency, very frequent in these patients [51]. Another important characteristic of pseudocereals is their lipid content, 2 to 3 times higher in comparison to common cereals like wheat. Cumulatively, these lipids are in its majority unsaturated which increases their nutritional quality [51]. Regarding vitamin B complex content, amaranth is a fine source of riboflavin; quinoa of riboflavin, thiamine, and folic acid; and buckwheat of thiamine, riboflavin, and pyridoxine [51]. Considering these nutrients are often in insufficient levels in CD patients, these pseudocereals are a viable option to include in a GFD.

The mineral deficit associated to a GFD can be surpassed and its quality improved through the substitution of processed foods of low nutritional value by pseudocereals of high nutritional value or through mineral supplementation [34]. Pseudocereals are a relevant source of calcium, iron, and zinc [51], particularly amaranth with a high calcium content, beneficial to CD patients who are predisposed to some degree of osteopenia and osteoporosis [50].

According to the National Nutrient Database for Standard Reference [53], by comparing raw values of pseudocereals to conventional wheat per 100 g, it is possible to verify that the latter is distinctive only because of the selenium values which are significantly higher than those of pseudocereals. However, in terms of protein levels, buckwheat, quinoa, and amaranth have approximately the double amount relative to wheat. In addition, all pseudocereals have a higher fiber content than wheat, especially bulgur and buckwheat. Regarding mineral content, amaranth has higher calcium, iron, and magnesium content than wheat and remaining pseudocereals (Table 1). Nevertheless, not even wheat nor the abovementioned pseudocereals have B12 since it is a vitamin of animal source.

Pseudocereals not only are sources of bioactive compounds not included in the nutrient definition, as some of its vitamins, minerals, peptides, and fatty acids also exhibit bioactive properties [54–56]. These include saponins, polyphenols, phytosterols, phytosteroids, and betalains, whose anti-inflammatory, antioxidant, anticancer, and antidiabetic activities are being unveiled and increasingly acknowledged as relevant [50, 56]. Phenolic compounds are among the most common and studied phytochemicals found in pseudocereals and seem to account (along with pseudocereals' nutritional richness) for the health benefits generally attributed to this food group [50, 57]. Metabolomic analysis of a variety of gluten-free flours showed a wide diversity of phenolic compounds, with flavonoids (anthocyanins, flavones, flavanones, isoflavonoids, flavonols, and flavanols), phenolic acids (hydroxycinnamics, hydroxybenzoics, and hydroxyphenylacetics), and tyrosol derivatives, being the most representative. Buckwheat contains the highest amount of total phenolics [275,5 mg of gallic acid equivalents (GAE)/100 g], followed by quinoa (130.2 mg GAE/100 g), and the lowest in amaranth (57.0 mg GAE/100 g). Similar variations for antioxidant capacity (determined *in vitro*) were also observed, 494,2 *μ*mol GAE/100 g for buckwheat, 289.6 *μ*mol GAE/100 g for black quinoa, and 76.2 *μ*mol GAE/100 g for amaranth, respectively [58].  Table 2 presents some of the most important bioactive components identified so far in buckwheat, quinoa, and amaranth. For an extensive review, please refer to [50, 56].

Although pseudocereals richness in nutrients and bioactive compounds is undisputed, it is also pertinent to mention the presence of antinutritional components, mainly phytic acid, saponins, and tannins. These can interfere with nutrients, preventing their proper digestion, absorption, or utilization. However, processing treatments such as germination, fermentation, puffing, and cooking improve pseudocereals nutritional (and organoleptic) properties and reduce these antinutrients, thus incrementing the availability or digestibility of nutrients [56, 59–61]. Combining dry roasting and fermentation of quinoa grains significantly degrades phytate and improves the estimated zinc and iron bioavailability. Fermentation using lactic acid bacteria can improve the nutritional and functional quality of pseudocereals, particularly by decreasing antinutritional factors like phytic acid, increasing phenolic compounds, and producing nutritional ingredients such as B-group vitamins [60]. Additionally, many studies found that fermentation of pseudocereals by *Lactobacillus* strains reduces the number of pathogenic microorganisms, like *Clostridioides* or *Escherichia,* and increases the synthesis of short-chain fatty acids, considered good sources for symbiotic formulations with potential application for dysbiosis, inflammatory bowel disease, obesity, and CD [61].

Although many studies (with cellular and animal models and humans) regarding pseudocereals have highlighted their health potential, standing out as the most consensual properties their antioxidant, antilipidemic, antihypertensive, and antidiabetic effects [50, 56], less evidence exists regarding interventional studies with CD patients. One such study evaluated the effect of eating quinoa (50 g) during 6 weeks, in adult CD patients (*n* = 19, 2 males and 17 females, with a median age of 59 years, BMI of 23 kg/m^2^, 9 years under a GFD). Participants were free to choose the cooking method but were recommended to consume quinoa flakes for breakfast as porridge or pancakes and quinoa grains prepared as rice or cooked in soups or stews. Gastrointestinal parameters (diarrhoea, abdominal pain, increased bowel movements, and vomiting) were self-reported throughout the study. Ten patients did not report any symptoms, while nine reported symptoms ranging from mild to moderate severity during the first 2 weeks (which seem to be related to the increase in diet's fibre) and one patient reported mild vomiting. Histological status of intestinal biopsies was assessed in 10 participants, being maintained or improved after quinoa consumption. Regarding blood biochemistry, a mild hypocholesterolemic effect was observed, with a slight reduction of total cholesterol, low-density lipoprotein, triglycerides, and high-density lipoprotein (but with values being only significant for the last one). As so, the authors concluded that short term consumption of quinoa is safe for CD individuals since it is well tolerated, does not exacerbate the disease, and may have a mild hypocholesterolemic effect [62].

Another study conducted a randomised cross-over trial, to assess the effect of buckwheat products on intestinal/extra-intestinal symptoms and biochemical parameters in 19 patients with Non-Celiac Gluten Sensitivity (NCGS). Participants (18 women and 1 man, median age of 44 years, mean BMI of 23.7 kg/m^2^) were randomly assigned to the experimental or control phases. During the experimental period, patients substituted all gluten-free products, for buckwheat products (pasta, hard tacks, biscuits, and buckwheat-flakes). Within the experimental phase, participants received (per day), 80 g of pasta, 60 g of hard tacks, 40 g of biscuits, and 50 g of flakes. During the control period, patients maintained their normal GFD. At the end of the first 6-week intervention or control phases, patients crossed over to the other treatment condition for the remaining 6 weeks of the study. Regarding gastrointestinal symptoms, patients experienced a significant decrease in the severity of abdominal pain and bloating during the experimental phase and, in an opposite way, a significant worsening trend for the majority of NCGS symptoms such as nausea, headache, joint/muscle pain, and attention disorders during the control period. On the other hand, during the intervention phase, a significant increase in serum magnesium and a significant reduction in the circulating levels of some pro-inflammatory cytokines (interferon gamma and monocyte chemotactic protein-1) were observed. Although these results support a health benefit of buckwheat products inclusion on NCGS individuals GFD, it is important to mention that among the buckwheat products tested in this study, only pasta was 100% buckwheat, as the other items also contained whole rice flour, corn flour, almonds flour, quinoa flour, and flaxseeds, which may also have contributed for the positive findings [63].

Regarding amaranth ingestion, the tolerability and effectiveness of amaranth products inclusion on gluten intolerant children (*n* = 37; aged from 1 year to 17 years) following a long GFD therapy were evaluated. After 9 to 12 months of intervention, an improvement of participants nutritional status was observed. Namely, underweight children decreased from 16.25 to 10.8% and children with short stature decreased from 10.8 to 5.4%. Additionally, biochemical analysis revealed that the initial abnormal low level of ionized calcium in the blood serum decreased from 37.8 to 10.8%. Furthermore, normalization of decreased blood serum levels of iron, copper, and zinc was observed in all patients who had a deficiency of these trace elements (in 13.5%, 8%, and 16.2% of children, respectively). Authors mentioned that amaranth products tested were well tolerated and did not report any allergic or dyspeptic reactions [64].

Therefore, though a GFD can be restrictive, there is another naturally gluten-free cereal group or pseudocereals that possess higher contents of protein, fiber, iron, magnesium, and other bioactive compounds, such as phenolics, among others. If introduced into a GFD, pseudocereals can diminish the most common nutritional deficiencies in CD patients, apart from providing a wider food choice and a less monotonous diet for the CD patients [34]. Although interventional studies with celiac individuals are very scarce, pseudocereals intake seems safe, well tolerated, and may contribute to improve symptomatology management.

## 5. Conclusion

Celiac disease is a growing health condition that affects primarily the small intestine due to the direct toxicity triggered by the ingestion and digestion of gluten. This pathology can originate significant nutritional deficiencies in vitamin B6 and B12, folate, vitamin D, iron, calcium, zinc, and copper. Until now, the only treatment available for CD is the rigorous implementation of a GFD in order to allow the full recovery of the intestinal mucosa and improve nutrient absorption. When choosing gluten-free food, specially processed one, it is crucial to look at the macronutrient profile, namely, the high content in saturated fat and low content in fiber and micronutrients which are prevalent in these types of food.

The incorporation of pseudocereals by themselves or as ingredients used by the food industry in gluten-free product development is a relevant alternative because of their richness in proteins, fiber, unsaturated fat, B complex vitamins, minerals like calcium, iron, magnesium, and other bioactive compounds. It can improve the nutritional status, well-being and health of CD patients and possibly expand their social inclusion in access to more food products and restaurants.

Finally, nutritionists also have a preponderant role in the dietary education of CD patients by encouraging them to prepare their meals with high quality gluten-free ingredients for a finer nutritional quality diet and a minor reliance on low nutritional value packaged food. They should also emphasize the importance of using food guides to manage dietary patterns and understanding basic concepts of food labeling. This would improve food literacy of CD patients and consequently lead to more favorable outcomes in terms of nutritional status and health.

## Figures and Tables

**Table 1:** Comparison of micronutrient composition between wheat and pseudocereals (in the National Nutrient Database for Standard Reference).

Cereal/pseudocereal	Protein (g)	Fiber (g)	Calcium (mg)	Iron (mg)	Magnesium (mg)	Selenium (*μ*g)	Vitamin B12 (*μ*g)
Wheat	7.49	1.1	28	2.14	82	42.5	0
Buckwheat	13.25	10	18	2.2	231	8.3	0
Quinoa	14.12	7	47	4.57	197	8.5	0
Millet	11.02	8.5	8	3.01	114	2.7	0
Bulgur	12.29	12.5	35	2.46	164	2.3	0
Sorghum	10.62	6.7	13	3.36	165	12.2	0
Amaranth	13.56	6.7	159	7.61	248	18.7	0

**Table 2:** Bioactive compounds present in amaranth, buckwheat, and quinoa (see Thakur et al. [56]).

Pseudocereals	Bioactive compounds	Examples
Buckwheat	Flavonoids	rutin (quercetin-3-rutinosid)
	Phenolic acids	p-hydroxyl benzoic syringic acid; vanillic acid; gallic acid; protocatechuic acid; ferulic acid; p-coumaric acid
Quinoa	Flavonoids	Quercetin; kaempferol; myricetin; isorhamnetin
	Phenolic acids	ferulic acid-4-glucoside
	Betalains	betanin
Amaranth	Flavonoids	Isoquercetin; rutin; nicotiflorine
	Betalains	Amaranthine; betacyanins
	Carotenoids	Zeaxanthin; lutein; *β*-carotene
